# Metabolic Engineering of *Escherichia coli* for Hyperoside Biosynthesis

**DOI:** 10.3390/microorganisms10030628

**Published:** 2022-03-16

**Authors:** Guosi Li, Fucheng Zhu, Peipei Wei, Hailong Xue, Naidong Chen, Baowei Lu, Hui Deng, Cunwu Chen, Xinjian Yin

**Affiliations:** 1 Anhui Engineering Laboratory for Conservation and Sustainable Utilization of Traditional Chinese Medicine Resources, Department of Biological and Pharmaceutical Engineering, West Anhui University, Lu’an 237012, China; 02000159@wxc.edu.cn (G.L.); 02000138@wxc.edu.cn (F.Z.); 02000148@wxc.edu.cn (P.W.); hsjx@wxc.edu.cn (N.C.); 02000171@wxc.edu.cn (B.L.); 02000107@wxc.edu.cn (H.D.); 2 Key Laboratory of Biomass Chemical Engineering of Ministry of Education, College of Chemical and Biological Engineering, Zhejiang University, Hangzhou 310027, China; 11428043@zju.edu.cn; 3 School of Marine Science, Sun Yat-sen University, Zhuhai 519080, China

**Keywords:** hyperoside, quercetin, UDP-dependent glycosyltransferase, UDP-glucose, metabolic engineering

## Abstract

Hyperoside (quercetin 3-*O*-galactoside) exhibits many biological functions, along with higher bioactivities than quercetin. In this study, three UDP-dependent glycosyltransferases (UGTs) were screened for efficient hyperoside synthesis from quercetin. The highest hyperoside production of 58.5 mg·L^−1^ was obtained in a recombinant *Escherichia coli* co-expressing UGT from *Petunia hybrida* (PhUGT) and UDP-glucose epimerase (GalE, a key enzyme catalyzing the conversion of UDP-glucose to UDP-galactose) from *E. coli*. When additional enzymes (phosphoglucomutase (Pgm) and UDP-glucose pyrophosphorylase (GalU)) were introduced into the recombinant *E. coli*, the increased flux toward UDP-glucose synthesis led to enhanced UDP-galactose-derived hyperoside synthesis. The efficiency of the recombinant strain was further improved by increasing the copy number of the PhUGT, which is a limiting step in the bioconversion. Through the optimization of the fermentation conditions, the production of hyperoside increased from 245.6 to 411.2 mg·L^−1^. The production was also conducted using a substrate-fed batch fermentation, and the maximal hyperoside production was 831.6 mg·L^−1^, with a molar conversion ratio of 90.2% and a specific productivity of 27.7 mg·L^−1^·h^−1^ after 30 h of fermentation. The efficient hyperoside synthesis pathway described here can be used widely for the glycosylation of other flavonoids and bioactive substances.

## 1. Introduction

Flavonoids (or bioflavonoids) are a class of plant and fungus secondary metabolites that play important roles as UV-B protectants, signaling molecules, and regulators of auxin transport [1,2,3]. Glycosylated flavonoids are the main derivatives of flavonoids. The glycosylation of flavonoids not only improves their solubility but also imparts stability, selectivity, and pharmacological activities [4,5]. 

Hyperoside (quercetin 3-*O*-galactoside), a type of flavonoid-*O*-glycoside, has gained a lot of attention as a valuable product for the pharmaceutical industry, e.g., as a powerful antioxidant with cytoprotective effects [6,7], or to inhibit the viruses hepatitis B [8] and SARS [9]. Furthermore, it has been attributed with anti-inflammatory [10], antidepressant [11,12], antifungal [13] and apoptotic [14] activities, rendering it an interesting therapeutic resulting in a steadily increasing market demand. At present, hyperoside is commercially purified from *Hypericum perforatum* L. or *Zanthoxylum bungeanum* by several steps, such as solvent extraction, column chromatography, and crystallization [15,16]. However, the process is complex, time-consuming, and thus unsuitable for large-scale production. The fermentation of metabolically engineered plant cells can be used to improve the production and overcome the low yields [17,18,19], but the process is still in its infancy, and is often restricted to small-scale production. 

A possible pathway for the preparation of hyperoside is through the glycosylation of quercetin at the 3C-O position. The chemical methods applied in the synthesis of flavonoid glycosides are limited due to side reactions, additional steps, environmental pollution, and low efficiency As an alternative, bioconversion catalyzed by UDP-glycosyltransferases (UGTs) has been explored as an alternative way to glycosylate flavonoids in vivo [20,21,22,23]. Recombinant strains have been constructed by overexpressing a UGT specific for quercetin and increasing the intracellular pool of UDP-galactose [24,25,26]. To date, the highest production of hyperoside reached 940.0 mg·L^−1^ in *E. coli,* with a conversion of 40.9% [26]. Despite the high production, the conversion still needs to be improved. Improving production and conversion through metabolic engineering requires the reconstruction of optimal biosynthetic pathways in *E. coli* because the gene sources, gene dose, enzyme expression levels, enzyme activities and special enzyme properties will all affect the metabolic flux and yield. In the present study, the screening of appropriate UGTs and the reconstruction of an efficient UDP-galactose synthetic pathway represent key factors involved in the improvement of hyperoside production and conversion [27,28,29].

UDP-galactose can be synthesized in situ from UTP and galactose-1-phosphate using UDP-sugar pyrophosphorylase (AtUSP) from *Arabidopsis* [30,31]. However, this process requires additional enzymes in order to construct a UTP regeneration system for the synthesis of UTP and an ATP regeneration system for the synthesis of galactose-1-phosphate. Considering that UDP-glucose is the precursor for UDP-galactose synthesis, enhancing UDP-glucose synthesis is a possible alternative to provide the UDP-galactose with high efficiency. Two methods have been reported to enhance the synthesis of UDP-glucose in *E. coli*. One is the overexpression of two key enzymes (phosphoglucomutase (Pgm) and UDP-glucose pyrophosphorylase (GalU)) in *E. coli* [31], and the other is the reconstruction of a novel UDP-glucose synthesis pathway via the simultaneous expression of Basp (sucrose phosphorylase from *Bifidobacterium adolescentis*) and UgpA (uridylyltransferase from *Bifidobacterium bifidum*) [26]. Both methods can use inexpensive and sustainable carbon sources (glucose or sucrose) to synthesize UDP-glucose.

**Figure 1:**
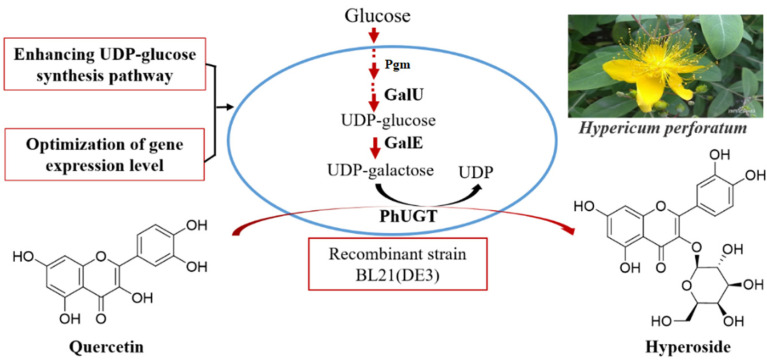
Synthesis of Hyperoside using recombinant *E. coli*. Pgm, phosphoglucomutase; GalU, UDP-glucose pyrophosphorylase; GalE, UDP-glucose 4-epimerase; PhUGT, UDP-dependent glycosyltransferases from *Petunia hybrida*.

In this study, three UGTs were first screened for hyperoside synthesis, following which the UDP-galactose synthetic efficiency in *E. coli* BL21(DE3) was enhanced by introducing GalE, Pgm, and GalU (Figure 1). The efficiency of the recombinant strain was further improved by increasing the copy number of PhUGT, which is a limiting step in the bioconversion. Finally, the fermentation conditions for hyperoside production with the final recombinant strain were determined.

## 2. Results

### 2.1. Screening UGTs for Hyperoside Production

**Figure 2:**
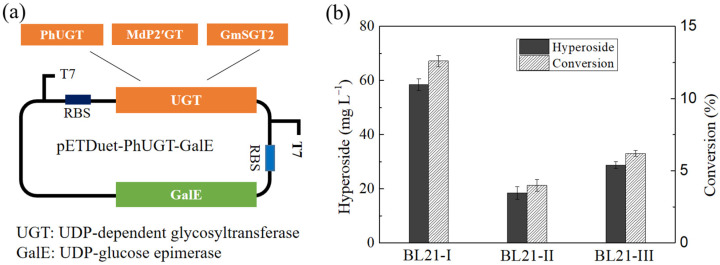
The construction and evaluation of recombinant *E. coli*. (**a**) The construction of the co-ex pressed strain with the first T7 promotor, followed by UGT (PhUGT, MdP2′GT, or GmSGT2), and the second T7 promotor, followed by GalE. (**b**) The performance of recombinant *E. coli* with 302.2 mg·L^−1^ quercetin.

The glycosylation of quercetin requires a highly efficient glycosyltransferase. It has been reported that PhUGT from *Petunia hybrida* (accession number AAD55985.1) [24,32], MdP2′GT from *Malus domestica* (accession number AMA68117.1) [33], and GmSGT2 from *Glycine max* (accession number BAI99584.1) [34] are capable of catalyzing the glycosylation of quercetin at the 3C-O position. The sequence alignment showed that the amino acid homology among these UGTs was very low (Figure S1), suggesting that they may have different catalytic properties for quercetin glycosylation. In order to improve their expression levels, PhUGT, MdP2′GT, and GmSGT2 were codon-optimized and cloned into pET-28a (+). However, UDP-glucose epimerase (GalE), an enzyme that catalyzes the reversible conversion of abundantly available UDP-glucose to UDP-galactose, is absent in *E. coli* BL21 (DE3), and this strain could not accumulate UDP-galactose. Thus, the glycosylation of quercetin using recombinant *E. coli* BL21 (DE3) harboring pET28a-PhUGT, pET28a-MdP2′GT, or pET28a-GmSGT2 did not produce hyperoside (data not shown). Therefore, the *GalE* gene was cloned from *E. coli* MG1655 and co-expressed with glucosyltransferase genes using pETDuet-1 (Figure 2a). The highest production of hyperoside was obtained in the recombinant strain BL21-I harboring pETDuet-PhUGT-GalE, i.e., 58.5 mg·L^−1^ with a molar conversion ratio of 12.6%, which was much higher than BL21-II harboring pETDuet-MdP2′GT-GalE, and BL21-III harboring pETDuet-GmSGT2-GalE (Figure 2b). These data also indicate that PhUGT is superior for hyperoside production.

### 2.2. Engineering the UDP-Glucose Synthesis Pathway to Enhance Hyperoside Production

**Figure 3:**
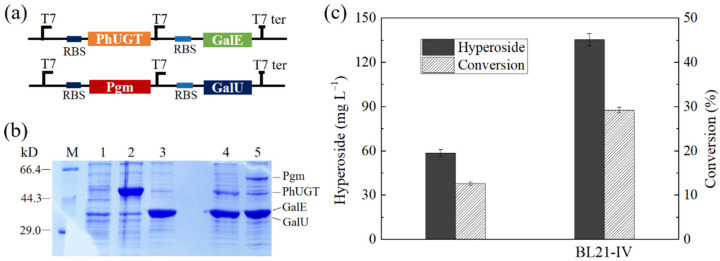
The construction and performance of BL21-IV. (**a**) The construction of BL21-IV harboring pETDuet-PhUGT-GalE with PhUGT and GalE, and pACYCDuet-Pgm-GalU with Pgm and GalU. (**b**) SDS-PAGE analysis of BL21-IV. M, Marker; Lane 1, BL21(DE3) with pET28a; Lane 2, BL21(DE3) with pET28a-PhUGT; Lane 3, BL21(DE3) with pET28a-GalE; Lane 4, BL21(DE3) with pETDuet-PhUGT-GalE; Lane 5, BL21(DE3) with pACYCDuet-Pgm-GalU. (**c**) The production of hyperoside in initial fermentation medium M-1 with 302.2 mg·L*^−^*^1^ quercetin.

UDP-glucose is the precursor of UDP-galactose synthesis, and its availability is a prerequisite for efficient hyperoside synthesis. UDP-glucose is a multigene, multipathway product; its synthetic pathway in *E. coli* is complex, and involves the glycolysis pathway, the pentose phosphate pathway, and nucleotide synthesis [35]. In the present study, in order to enhance the flux to UDP-glucose synthesis from glucose, two key enzymes (phosphoglucomutase (Pgm) and UDP-glucose pyrophosphorylase (GalU)) directly involved in the synthesis of UDP-glucose [36] were simultaneously overexpressed in BL21-I by pACYCDuet-1 (Figure 3a). SDS-PAGE analysis showed that PhUGT, GalE, Pgm, and GalU were successfully overexpressed in the resulting strain, BL21-IV, harboring both pETDuet-PhUGT-GalE and pACYCDuet-Pgm-GalU (Figure 3b). After 24 h of fermentation, BL21-IV produced 135.4 mg·L^−1^ hyperoside, which was 2.3 times higher than that in BL21-I (Figure 3c). The results indicated that by overexpressing a phosphoglucomutase and a UDP-glucose pyrophosphorylase, BL21-IV has the capacity to accumulate more UDP-galactose for the synthesis of hyperoside. 

### 2.3. Enhacing the PhUGT Expression Level to Improve Hyperoside Synthesis

**Figure 4:**
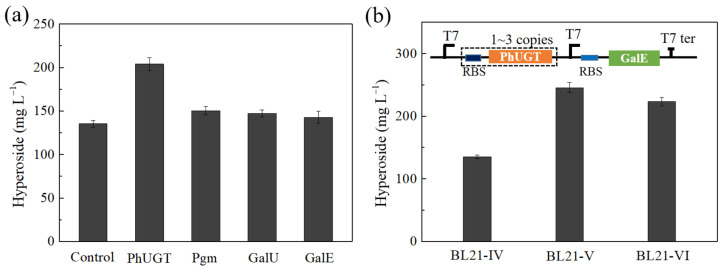
Optimization of the expression of PhUGT in the co-expression strain by different copies. (**a**) Hyperoside production by strain BL21-IV with the exogenous addition of 200 mU·mL^−1^ PhUGT, Pgm, GalU, or GalE. (**b**) The production of hyperoside in the co-expression strain with different copies of PhUGT. BL21-IV, one copy; BL21-V, two copies; BL21-VI, three copies.

The precise expression of multiple genes in *E. coli* is critical for the successful heterologous biosynthesis of valuable compounds. In order to find the bottleneck for the synthesis of hyperoside, whole cell catalysts of BL21-PhUGT, BL21-Pgm, BL21-GalU, and BL21-GalE were added to the fermentation medium. The hyperoside production increased by 49.9% when 200 mU·mL^−1^ PhUGT was added, whereas it increased by 11.1%, 8.7%, and 5.4% when the same amounts of Pgm, GalU and GalE were added, respectively (Figure 4a). This indicates that PhUGT acts as a limiting step. In this study, we attempted to improve the glycosylation efficiency by increasing the gene copy number of PhUGT to two (BL21-V) and three (BL21-VI) (Figure 4b). As expected, the increase in the copy number dramatically enhanced the accumulation of hyperoside (Figure 4a). The BL21-V in initial fermentation medium M-1 produced 245.6 mg·L^−1^ hyperoside from 302.2 mg·L^−1^ quercetin, corresponding to a 52.9% conversion rate (Figure 4b). Interestingly, the hyperoside titer and conversion were lower in BL21-VI than in BL21-V. It seems that the duplication of the PhUGT gene improved its activity while also affecting the expression of Pgm, GalU, or GalE, creating insufficient UDP-galactose for further conversion.

### 2.4. Optimizing the Fermentation Conditions

**Figure 5:**
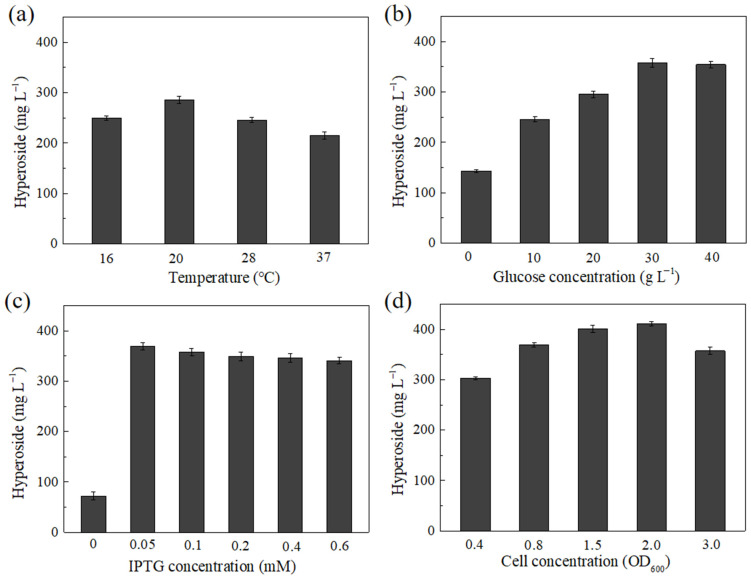
Optimization of hyperoside fermentation conditions using recombinant strain BL21-V. (**a**) The effects of the induction temperature on hyperoside production. (**b**) The effects of the glucose concentration on hyperoside production. (**c**) The effects of the IPTG concentration on hyperoside production. (**d**) The effects of the cell concentration on hyperoside production.

**Figure 6:**
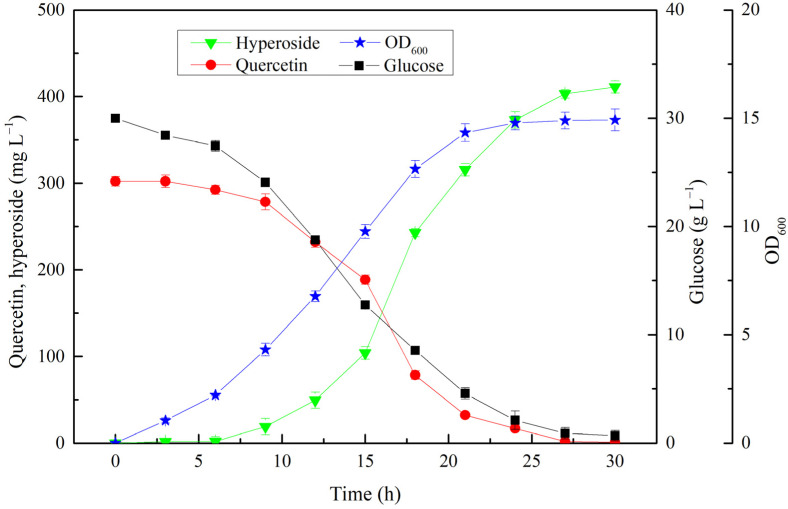
Time course analysis of hyperoside production under optimal fermentation conditions using recombinant strain BL21-V.

The fermentation conditions were optimized in shake flasks using recombinant strain BL21-V. Induction at low temperatures could reduce the inclusion body formation for some proteins. The optimal induction temperature for hyperoside was 20 °C (Figure 5a). The hyperoside production at this induction temperature was 285.3 mg·L^−1^, which was 32.5% and 16.1% higher than that at 37 °C and 28 °C, respectively. Glucose provided UDP-glucose to as a sugar donor, and was also used as the carbon source. The production of hyperoside was remarkably improved by the addition of glucose. The maximal hyperoside production reached 358.1 mg·L^−1^ for the initial medium containing 3.0% glucose after 24 h of fermentation (Figure 5b). IPTG and cell concentrations can affect the relationship between the expression of recombinant proteins and strain growth. In this study, the optimal IPTG and cell concentrations were 0.05 mM and OD_600_ = 2.0, respectively (Figure 5c,d). The time course analysis of hyperoside production and cell growth during the cultivation of BL21-V was also performed. As shown in Figure 6, the OD_600_ reached its maximum of 14.9 after 24 h, and the hyperoside production reached a maximum of 411.2 mg·L^−1^ after 30 h, with a molar conversion ratio of 88.5%. 

### 2.5. Hyperoside Production by Substrate-Fed Batch Fermentation

**Figure 7:**
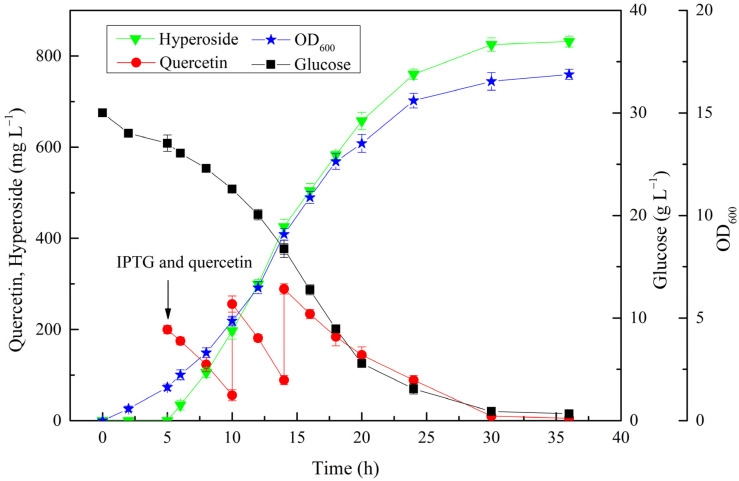
Hyperoside production by substrate-fed batch fermentation.

In order to obtain an even higher hyperoside production, 600.0 mg·L^−1^ quercetin and 0.05 mM IPTG were fed to the medium at an induction temperature of 20 °C when the OD_600_ of the cells reached 2.0. Considering that a high concentration of quercetin might inhibit cell growth, 200.0 mg·L^−1^ quercetin was fed every time. The time courses of the glucose and quercetin consumption, cell growth, and hyperoside production are shown in Figure 7. The cell densities steadily increased over time, and reached a maximum OD_600_ of 16.9 at 30 h. The hyperoside production continued to increase along with the cell growth, whereas the productivity varied with the aging of the cells and increased hyperoside concentration. The specific productivity was 32.9 mg·L^−1^·h^−1^ after 6 h of induction, and it increased to 57.0 mg·L^−1^·h^−1^ in the second periods but gradually decreased to 25.4 mg·L^−1^·h^−1^ in the third period. Finally, 831.6 mg·L^−1^ hyperoside was produced within 30 h, with a specific productivity of 27.7 mg·L^−1^·h^−1^ and a conversion ratio of 90.2%. 

## 3. Materials and Methods

### 3.1. Strains, Media, Plasmids, and Chemicals

*E. coli* strains DH5α and BL21 (DE3) were used for the construction of the plasmids and as a host strain for hyperoside production. The strains were grown at 37 °C in Luria-Bertani with certain antibiotics. Medium M-1 contained 10 g·L^−1^ tryptone, 5 g·L^−1^ yeast extract, and 10 g·L^−1^ NaCl; 10 g·L^−1^ glucose was used for the hyperoside production. Medium M-2 contained 10 g·L^−1^ tryptone, 5 g·L^−1^ yeast extract, and 10 g·L^−1^ NaCl; 30 g·L^−1^ glucose was used for substrate-fed batch fermentation. The gene overexpression was performed using pET28a(+), pACYCDuet-1, and pETDuet-1 (Novagen, Darmstadt, Germany) as expression vectors. The restriction enzymes T4 DNA ligase and PrimeSTAR^@^HS DNA polymerase were purchased from Takara (Dalian, China). All of the chemicals were purchased from Sigma Aldrich, Sangon Biotech, Aladdin, Macklin or TCI Chemicals.

### 3.2. Plasmid Construction

*PhUGT* (accession number AF165148.1), *MdP2′GT* (accession number KT444675.1), *GmSGT2* (accession number AB473730.1), and *GmSUS* (accession number NM_001250596.2) were codon-optimized and synthesized into pET28a between *Noc* I and *Hind* III (Sangon Biotech, Shanghai, China) in order to obtain pET28a-PhUGT, pET28a-MdP2′GT, pET28a-GmSGT2, and pET28a*-GmSUS* (Table 1)*. Pgm*, *GalU, and GalE* were amplified from the genome of *E. coli* MG1655 with the primers Pgm-F and Pgm-R, GalU-F and GalU-R, and GalE-F and GalE-R (Table S1). The resulting PCR products were inserted into the expression vector pET28a between *Noc* I and *Hind* III according to the standard One Step Cloning Kit (Vazyme Biotech, Nanjing, China) to create recombinant plasmids pET28a-GalE, pET28a-Pgm, and pET28a-GalU (Table 1).

*PhUGT*, *MdP2′GT*, and *GmSGT2* were amplified using pET28a-PhUGT, pET28a-MdP2′GT, pET28a-GmSGT2 as templets with the primers PhUGT-mcs1-F and PhUGT-mcs1-R, MdP2′GT-mcs1-F and MdP2′GT-mcs1-R, and GmSGT2-mcs1-F and GmSGT2-mcs1-R (Table S1), and the fragments were inserted into *MCS*1 of pETDuet-1 between *Nco* I and *Hind* III according to the standard One Step Cloning Kit (Vazyme Biotech, Nanjing, China), resulting in pETDuet-PhUGT, pETDuet-MdP2′GT, and pETDuet-GmSGT2. *GalE* was amplified from the genome of *E. coli* MG1655 with the primers GalE-mcs2-F and GalE-mcs2-R (Table S1), and the fragments were inserted into *MCS*2 of pETDuet-PhUGT, pETDuet-MdP2′GT, and pETDuet-GmSGT2 between *Nde* I and *Xho* I to obtain pETDuet-PhUGT-GalE, pETDuet-MdP2′GT-GalE, and pETDuet-GmSGT2-GalE (Table 1) using the same method described above. 

*PhUGT(2)* and *PhUGT(3)* (*PhUGT* with an added RBS sequence) were obtained by PCR using PhUGT(2)-mcs1-F and PhUGT(2)-mcs1-R, and PhUGT(3)-mcs1-F and PhUGT(3)-mcs1-R (Table S1) as primers, and pET28a-PhUGT as a templet. The *PhUGT(2)* fragments were inserted into pETDuet-PhUGT-GalE at restriction site *Hind* III using T4 DNA ligase, resulting in pETDuet-PhUGT(2)-GalE (Table 1). The *PhUGT(3)* fragments were inserted into pETDuet-PhUGT(2)-GalE at restriction site *Not* I using T4 DNA ligase, resulting in pETDuet-PhUGT(2)-GalE (Table 1). *Pgm* and *GalU* were amplified from the genome of *E. coli* MG1655 with the primers Pgm-mcs1-F and Pgm-mcs1-R, and GalU-mcs2-F and GalU-mcs2-R (Table S1), and the two fragments were inserted into *MCS1* of pACYCDuet-1 between *Nco* I and *Hind* III, and *MCS*2 of pACYCDuet-1 between *Nde* I and *Xho* I, resulting in pACYCDuet-Pgm-GalU. 

### 3.3. Hyperoside Production Using Recombinant Strains

In order to compare the hyperoside production by recombinant strains harboring different UGT genes, the plasmids pET28a-PhUGT, pET28a-MdP2′GT, pET28a-GmSGT2, pETDuet-PhUGT-GalE, pETDuet-MdP2′GT-GalE, and pETDuet-GmSGT2-GalE were transformed into *E. coli* BL21(DE3), respectively, to obtain the recombinant strains BL21-PhUGT, BL21-MdP2′GT, BL21-GmSGT2, BL21-I, BL21-II, and BL21-III (Table 1). The plasmids pETDuet-PhUGT-GalE and pACYCDuet-Pgm-GalU were simultaneously transformed into *E. coli* BL21(DE3) to obtain BL21-IV (Table 1). The plasmids pETDuet-PhUGT(2)-GalE and pACYCDuet-Pgm-GalU were simultaneously transformed into *E. coli* BL21(DE3) to obtain BL21-V (Table 1). The plasmids pETDuet-PhUGT(3)-GalE and pACYCDuet-Pgm-GalU were simultaneously transformed into *E. coli* BL21(DE3) to obtain BL21-VI (Table 1). 

The recombinant strains were inoculated into 5 mL M-1 medium containing certain antibiotics, and were grown at 37 °C until the absorbance at 600 nm reached 0.8. Quercetin and IPTG were added to final concentrations of 302.2 mg·L^−1^ and 0.1 mM, respectively. The induced cultures were cultured at 28 °C and 200 rpm for 24 h. Samples were withdrawn throughout the fermentation process, and five volumes of methanol were directly added to the fermentation broths and subjected to HPLC analysis. 

### 3.4. Determination of the Limiting Step of Hyperoside Production in BL21-IV Fermentation

The plasmids pET28a-Pgm, pET28a-GalU, and pET28a-GalE were transformed into *E. coli* BL21 (DE3), respectively, to obtain BL21-Pgm, BL21-GalU, and BL21-GalE (Table 1). Recombinant strains BL21-Pgm, BL21-GalU, BL21-GalE, and BL21-PhUGT were induced to express the recombinant enzymes by adding IPTG to a final concentration of 0.1 mM at an OD_600_ of approximately 0.8 using incubation at 20 °C for approximately 20 h. The recombinant cells (500 mL) were harvested by centrifugation at 5000× *g* for 10 min at 4 °C, washed twice with distilled water, and resuspended in 50 mL Tris-HCl buffer (pH 7.5). The cell extracts were then centrifuged (20,000× *g*, 4 °C, 30 min). The enzyme activity of the resulting supernatants was assayed, and 200 mU mL*^−^*^1^ of each enzyme was added to BL21-IV medium after 12 h fermentation. Samples were withdrawn and assayed by HPLC after 24 h of fermentation. 

### 3.5. Enzyme Activity

The glycosyltransferase activity was measured as described previously [37]. The activities of Pgm and GalU were measured as described previously [38,39]. The enzyme activity of GalE was measured in a two-step assay, as described previously [32].

### 3.6. Optimizing the Fermentation Conditions

The optimizing of the fermentation conditions was conducted using the recombinant strain BL21-V. The fermentations were performed in a 250 mL flask containing 50 mL of medium with appropriate antibiotics at an initial temperature of 37 °C. The effects of the induction temperature (16–37 °C), glucose concentration (0–40 g·L^−1^), IPTG concentration (0–0.6 mM) and cell concentration (OD_600_ = 0.4–3.0) on hyperoside production were determined.

### 3.7. Hyperoside Production by Substrate-Fed Batch Fermentation

Substrate-fed batch fermentations were carried out in a 1 L shake flask with a working volume of 100 mL containing 50 μg·mL^−1^ ampicillin and 34 μg·mL^−1^ chloramphenicol. M-2 medium was used for the biotin production. IPTG and quercetin were fed to a final concentration of 0.5 mM and 200 mg·L^−1^ when OD_600_ = 2.0. The cells were cultured at 20 °C after the feed of IPTG and quercetin. After the depletion of the quercetin added initially, a feeding solution containing 200 mg·L^−1^ quercetin was fed. Samples were withdrawn throughout the fermentation process and subjected to HPLC analysis. 

### 3.8. HPLC Analysis

The samples were analyzed by high-performance liquid chromatography (HPLC) (HP1100, Agilent 1100 series) equipped with a C18 column (5 μm, 4.6 × 250 mm, Agilent). The column temperature was maintained at 30 °C, and detection was carried out at 368 nm in 55% methanol with a flow rate of 0.6 mL·min^−1^. All of the assays were carried out in triplicate.

### 3.9. Product Purification and Structural Identification

The fermentation broths were created by centrifugation at 12,000× *g* for 10 min. The supernatant was then chromatographed on AB-8 column macroporous resin (2.5 × 30 cm). Distilled water and 10% ethanol were first used to elute the resin. Then, 50% ethanol was used to elute the hyperoside, and the elution fractions were evaporated and determined using ^1^H-NMR and ^13^C-NMR (Bruker AVANCE IIII 400), using DMSO-*d_6_* as the solvent. ^1^H NMR (400 MHz, DMSO-*d_6_*): 12.58 (s, 1H), 10.82 (s, 1H), 9.69 (s, 1H), 9.11 (s, 1H), 7.62 (dd, *J* = 1.8, 8.4 Hz, 1H, H-6′), 7.47 (d, *J* = 1.8 Hz, 1H, H-2′), 6.76 (d, *J* = 8.4 Hz, 1H, H-5′), 6.35 (d, *J* = 1.6 Hz, 1H, H-8), 6.15 (d, *J* = 1.6 Hz, 1H, H-6), 5.33 (d, *J* = 7.6 Hz, 1H, H-1″), 3.20–5.10 (11H). ^13^C NMR (400 MHz, DMSO-*d_6_*): 178.0 (C-4), 164.6 (C-7), 161.7 (C-5), 156.8 (C-2), 156.7 (C-9), 148.9 (C-4′), 145.3 (C-3′), 134.0 (C-3), 122.5 (C-6′), 121.6 (C-1′), 116.4 (C-5′), 115.6 (C-2′), 104.4 (C-10), 102.3 (C-1″), 99.1 (C-6), 94.0 (C-8), 76.3 (C-5″), 73.7 (C-3″), 71.7 (C-2″), 68.4 (C-4″), 60.6 (C-6″). The NMR spectroscopies are shown in Figures S2 and S3.

## 4. Discussion

The goal of this study was to develop a green and highly efficient method for the production of hyperoside. A possible pathway for the preparation of hyperoside is through the glycosylation of quercetin at the 3C-O position. To this end, three functionally identical genes from different organisms were tested in order to identify a compatible glucosyltransferase with high efficiency. Given that in vitro catalytic efficiency does not always correlate with in vivo biotransformation efficiency [40], we compared the final conversion of recombinant strains instead of the specific activity of each enzyme, and PhUGT from *Petunia hybrida* was finally selected for further study. 

The biosynthesis of UDP-galactose is a prerequisite to the formation of hyperoside. However, *E. coli* BL21 (DE3) cannot synthesize UDP-galactose because it lacks GalE, which can catalyze the reversible conversion of available UDP-glucose into UDP-galactose. It has been reported that UDP-galactose can be synthesized by UDP-sugar pyrophosphorylase from *Arabidopsis* using galactose-1-phosphate and UTP as substrates [30,31], but the process requires both ATP and UTP regeneration systems for the regeneration of galactose-1-phosphate and UTP. In this study, the supply of UDP-galactose in BL21 (DE3) was realized by introducing the *GalE* gene, resulting in 58.5 mg·L^−1^ hyperoside being obtained. However, the level of UDP-galactose in BL21 (DE3) was insufficient for higher hyperoside production. The supply of UDP-galactose can be further improved by enhancing the synthesis of UDP-glucose, the precursor of UDP-galactose. According to the literature, there are two methods that can be applied. One is the overexpression of two key enzymes (phosphoglucomutase (Pgm) and UDP-glucose pyrophosphorylase (GalU)) in *E. coli* [31], and the other is the reconstruction of a novel UDP-glucose synthesis pathway via the simultaneous expression of Basp (sucrose phosphorylase from *Bifidobacterium adolescentis*) and UgpA (uridylyltransferase from *Bifidobacterium bifidum*) [26]. In order to avoid the introduction of more heterologous proteins in the resulting recombinant *E. coli*, the first method was applied. This increased the hyperoside production from 58.5 to 135.4 mg·L^−1^, indicating that the engineering of the nucleotide pathway in *E. coli* is critical to increase the yield of flavonoid glycosides.

Although PhUGT has been proven to be an excellent enzyme for efficient hyperoside production, the catalytic activity of PhUGT became a limiting step when the UDP-galactose synthetic pathway was engineered in *E. coli*. Direct evolution or semi-rational protein engineering has been used to increase the catalytic efficiency of various UDP-dependent glycosyltransferases [41,42]. However, high-throughput screening is a bottleneck in glycosyltransferase engineering. In this study, we attempted to improve the glycosylation efficiency by simply increasing the copy number of PhUGT, and hyperoside production increased from 135.4 to 245.6 mg·L^−1^ when the copy number increased to two copies. Previous reports demonstrated that flavonoid glycoside production could be significantly affected by fermentation conditions [43]. In this study, various fermentation conditions were investigated, including the induction temperature, glucose, IPTG concentration, and timing of the induction. Under the optimal conditions, 411.2 mg·L^−1^ hyperoside was produced from 302.2 mg·L^−1^ quercetin, with a molar conversion ratio of 88.5%. In order to further improve the hyperoside production, a higher concentration of substrate should be used. Given that a high concentration of quercetin might inhibit cell growth [26,43], the production was conducted using substrate-fed batch fermentation, into which 200 mg·L^−1^ was fed every time. Finally, 831.6 mg·L^−1^ hyperoside was produced within 30 h, with a corresponding molar conversion of 90.2%. To date, the highest production of hyperoside reached 940.0 mg·L^−1^ in *E. coli,* with a conversion of only 40.9%. The high conversion obtained in the present study can contribute to an overall cost saving for large-scale production.

## 5. Conclusions

Functionally identical genes from different organisms were tested, and PhUGT from *P. hybrida* was found to be more effective than that from *Malus domestica* and *Glycine max*. The synthesis of hyperoside was realized by introducing the GalE gene into recombinant *E. coli,* resulting in 58.5 mg·L^−1^ hyperoside being obtained. The level of UDP-galactose was improved by enhancing the supply of UDP-glucose, via the overexpression of two key enzymes in the pathway (Pgm and GalU). This increased the hyperoside production from 58.5 to 135.4 mg·L^−1^. The glycosylation efficiency was further improved by increasing the copy number of PhUGT to two copies, resulting in the hyperoside titer being increased from 135.4 mg·L^−1^ to 245.6 mg·L^−1^. Under optimal fermentation conditions, 831.6 mg·L^−1^ hyperoside was produced within 30 h with a corresponding molar conversion of 90.2% in a substrate-fed batch fermentation. The efficient production of hyperoside may be a good model for the biosynthesis of other flavonoids and bioactive substances in *E. coli*.

## Figures and Tables

**Table 1:** Plasmids and strains used in the present study.

Plasmids or Strains	Relevant Properties or Genetic Marker	Source
Plasmids		
pET28a	f1 ori, Kan^r^	Novagen
pETDuet-1	f1 ori, Amp^r^	Novagen
pACYCDuet-1	P15A ori, Cm^r^	Novagen
pET28a-PhUGT	pET28a + *PhUGT* from *Petunia hybrida*	This study
pET28a-MdP2′GT	pET28a + *MdP2′GT* from *Malus domestica*	This study
pET28a-GmSGT2	pET28a + *GmSGT2* from *Glycine max*	This study
pET28a-GalE	pET28a + *GalE* from *E. coli MG1655*	This study
pET28a-Pgm	pET28a + *Pgm* from *E. coli MG1655*	This study
pET28a-GalU	pET28a + *GalU* from *E. coli MG1655*	This study
pETDuet-PhUGT-GalE	pETDuet-1 + *PhUGT* from *P. hybrida* + *GalE* from *E. coli MG1655*	This study
pETDuet-MdP2′GT-GalE	pETDuet-1 + *MdP2′GT* from *M. domestica* + *GalE* from *E. coli MG1655*	This study
pETDuet-GmSGT2-GalE	pETDuet-1 + *GmSGT2* from *G. max* + *GalE* from *E. coli MG1655*	This study
pETDuet-PhUGT(2)-GalE	pETDuet-1 + *PhUGT* (2 copies) from *P. hybrida* + *GalE* from *E. coli MG1655*	This study
pETDuet-PhUGT(3)-GalE	pETDuet-1 + *PhUGT* (3 copies) from *P. hybrida* + *GalE* from *E. coli MG1655*	This study
pACYCDuet-Pgm-GalU	pACYCDuet-1 + *Pgm* from *E. coli MG1655* + *GalU* from *E. coli MG1655*	This study
Strains		
BL21 (DE3)	F^−^ *ompT hsdSB*(r_B_^−^ m_B_^−^) *gal dcm lon* (DE3)	Novagen
BL21-PhUGT	BL21 (DE3) harboring pET28a-PhUGT	This study
BL21-MdP2′GT	BL21 (DE3) harboring pET28a- MdP2′GT	This study
BL21-GmSGT2	BL21 (DE3) harboring pET28a- GmSGT2	This study
BL21-I	BL21 (DE3) harboring pETDuet-PhUGT-GalE	This study
BL21-II	BL21 (DE3) harboring pETDuet-MdP2′GT-GalE	This study
BL21-III	BL21 (DE3) harboring pETDuet- GmSGT2-GalE	This study
BL21-IV	BL21 (DE3) harboring pETDuet-PhUGT-GalE and pACYCDuet-Pgm-GalU	This study
BL21-V	BL21 (DE3) harboring pETDuet-PhUGT(2)-GalE and pACYCDuet-Pgm-GalU	This study
BL21-VI	BL21 (DE3) harboring pETDuet-PhUGT(3)-GalE and pACYCDuet-Pgm-GalU	This study

## Data Availability

The data are contained within the article.

## Supplementary Materials

The following supporting information can be downloaded at: https://www.mdpi.com/article/10.3390/microorganisms10030628/s1. Figure S1: Multiple sequence alignment of PhUGT, MdP2′GT, and GmSGT2; Figure S2: ^13^C NMR spectra of purified hyperoside; Figure S3: ^13^C NMR spectra of purified hyperoside; Table S1: Primers used in the present study.
