# Supplementary material for: Metabolic Engineering of Escherichia coli for Hyperoside Biosynthesis
Source: Microorganisms. 2022 Mar 16;10(3):628. doi: 10.3390/microorganisms10030628 (PMC8949062; doi:10.3390/microorganisms10030628)
Supplement: Supplementary file 1 [file microorganisms-10-00628-s001.zip › microorganisms-1575016-supplementary.pdf]

Supporting Information

Metabolic Engineering of *Escherichia coli* for Hyperoside Biosynthesis

Guosi Li [1], Fucheng Zhu [1], Peipei Wei [1], Hailong Xue [2], Naidong Chen [1], Baowei Lu [1], Hui Deng [1], Cunwu Chen [1,*], Xinjian Yin [3,*]

[1] Anhui Engineering Laboratory for Conservation and Sustainable Utilization of Traditional Chinese Medicine Resources, Department of Biological and Pharmaceutical Engineering, West Anhui University, Lu'an, 237012, Anhui, China; 02000159@wxc.edu.cn (G.L.); fucheng323@163.com (F.Z.); 1209091181@qq.com (P.W.); 2004cnd@163.com; lu_baowei123@126.com; dhup@qq.com

[2] Key Laboratory of Biomass Chemical Engineering of Ministry of Education, College of Chemical and Biological Engineering, Zhejiang University, Hangzhou, 310027, China; 11428043@zju.edu.cn

[3] School of Marine Science, Sun Yat-sen University, Zhuhai, 519080, China

* Correspondence: chengcw@wxc.edu.cn (C.C.); yinxj5@mail.sysu.edu.cn (X.Y.)

List of Entries

Figure S1 Multiple sequence alignment of PhUGT, MdP2′GT, and GmSGT2……………………….3
Figure S2 ^{13}C NMR spectra of purified hyperoside. ……………………. …………………………3
Figure S3 ^{1}H NMR spectra of purified hyperoside. ……………………. …………………………..4

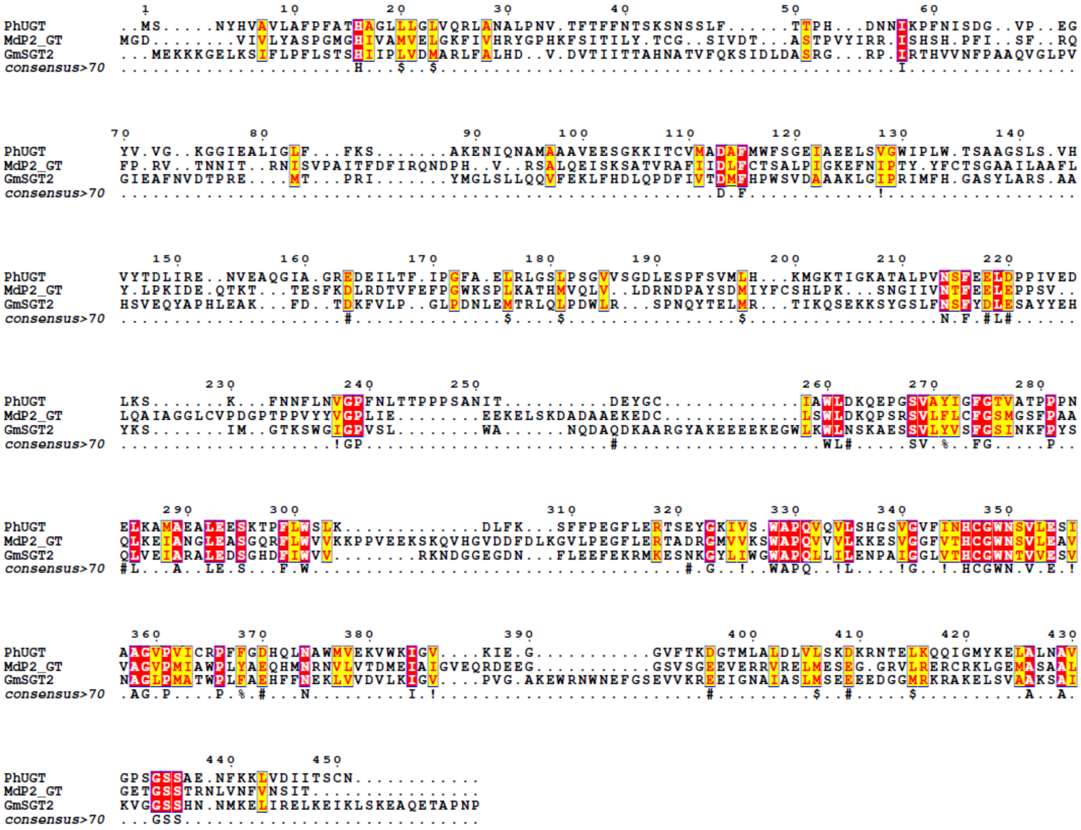

Figure S1. Multiple sequence alignment of PhUGT, MdP2′GT, and GmSGT2.

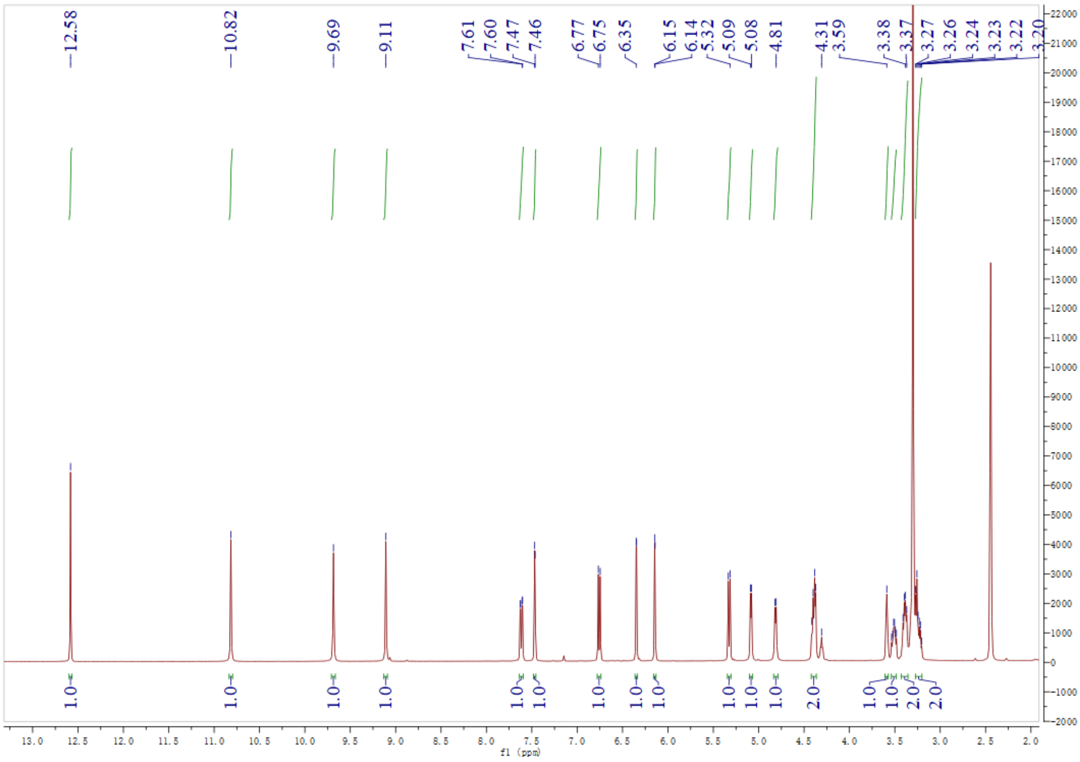

Figure S2. ^{13}C NMR spectra of purified hyperoside.

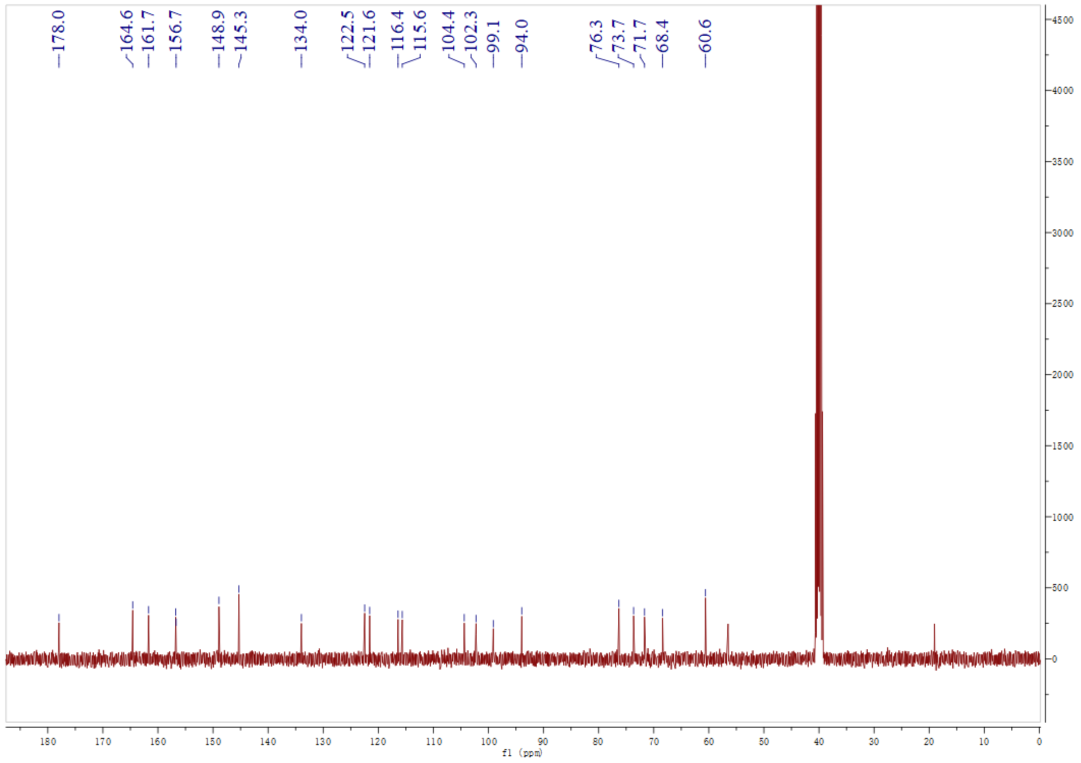

Figure S3. ^{1}H NMR spectra of purified hyperoside.
